# The epidemic dynamics of hepatitis C virus subtypes 4a and 4d in Saudi Arabia

**DOI:** 10.1038/srep44947

**Published:** 2017-03-21

**Authors:** Ahmed A. Al-Qahtani, Guy Baele, Nisreen Khalaf, Marc A. Suchard, Mashael R. Al-Anazi, Ayman A. Abdo, Faisal M. Sanai, Hamad I. Al-Ashgar, Mohammed Q. Khan, Mohammed N. Al-Ahdal, Philippe Lemey, Bram Vrancken

**Affiliations:** 1 Department of Infection and Immunity, King Faisal Specialist Hospital & Research Center, Riyadh, Saudi Arabia; 2 Department of Microbiology and Immunology, Alfaisal University School of Medicine, Riyadh, Saudi Arabia; 3 KU Leuven - University of Leuven, Department of Microbiology and Immunology, Rega Institute for Medical Research, B-3000 Leuven, Belgium; 4 Department of Biomathematics, David Geffen School of Medicine at UCLA, University of California, Los Angeles, USA; 5 Department of Human Genetics, David Geffen School of Medicine at UCLA, University of California, Los Angeles, USA; 6 Section of Gastroenterology, Department of Medicine, College of Medicine, King Saud University, Riyadh, Saudi Arabia; 7 Gastroenterology Unit, Department of Medicine, King Abdulaziz Medical City, Jeddah, Saudi Arabia; 8 Gastroenterology Unit, Department of Medicine, King Faisal Specialist Hospital & Research Center, Riyadh, Saudi Arabia

## Abstract

The relatedness between viral variants sampled at different locations through time can provide information pertinent to public health that cannot readily be obtained through standard surveillance methods. Here, we use virus genetic data to identify the transmission dynamics that drive the hepatitis C virus subtypes 4a (HCV4a) and 4d (HCV4d) epidemics in Saudi Arabia. We use a comprehensive dataset of newly generated and publicly available sequence data to infer the HCV4a and HCV4d evolutionary histories in a Bayesian statistical framework. We also introduce a novel analytical method for an objective assessment of the migration intensity between locations. We find that international host mobility patterns dominate over within country spread in shaping the Saudi Arabia HCV4a epidemic, while this may be different for the HCV4d epidemic. This indicates that the subtypes 4a and 4d burden can be most effectively reduced by combining the prioritized screening and treatment of Egyptian immigrants with domestic prevention campaigns. Our results highlight that the joint investigation of evolutionary and epidemiological processes can provide valuable public health information, even in the absence of extensive metadata information.

The hepatitis C virus (HCV) imposes a considerable burden on societies and health-care systems throughout the world, and currently infects an estimated 80 (64–103) million people^1^. Despite dramatic improvements in response rates to antiviral therapy following the introduction of highly potent direct-acting antivirals, their global impact on the HCV prevalence is limited^2^. Consequently, a thorough understanding of the dynamics of HCV spread remains important to guide public health efforts.

Because the evolutionary and epidemiological processes of HCV occur on the same time scale, viral genetic data can be used to elucidate transmission patterns using phylodynamic tools^3^,^4^. When clinical, demographic and/or behavioral metadata is available these tools can be used to identify populations at increased risk (e.g. Jacka, *et al*.^5^ for HCV). However, such richly annotated virus genetic data is usually not available, in which case researchers can often fall back on the more widely shared temporal and geospatial annotations to uncover epidemiologically relevant patterns of virus flow^6^,^7^,^8^.

HCV is classified into several genotypes and subtypes that are associated with varying geographical distributions. Some subtypes (e.g. 1a, 1b, 2a and 3a) are found abundantly across the globe, but the spread of others remains more confined to particular geographical areas^1^. An example of the latter is genotype 4, which is endemic in Central Africa from where multiple lineages were introduced into North Africa and the Middle East^9^. Among genotype 4 subtypes, subtype 4a (HCV4a) dominates the HCV epidemic in Egypt, which likely reflects the interplay between a founder effect and past unsafe injection practices during large public health campaigns^10^,^11^. Genotype 4 is also ubiquitously present in Saudi Arabia, a neighboring country of Egypt, where it accounts for 60% to 70% of the HCV infections^12^,^13^. Of these, about 50% can be attributed to HCV4a, and another ~40% are due to subtype 4d (HCV4d)^13^. While HCV4a is omnipresent in Egypt, only few HCV4d variants circulate in this country^12^. This suggests that whereas the HCV4a epidemics in Egypt and Saudi Arabia may overlap, HCV4d may have been introduced from elsewhere, or constitutes a more confined local epidemic.

Despite the substantial genotype 4 burden in Saudi Arabia, its population level patterns of spread have not been investigated to date. Here we make use of newly generated and publicly available virus genetic data to, for the first time, formally characterize the transmission dynamics of the Saudi Arabia HCV4a and HCV4d epidemics, with a focus on the spatial (HCV4a) and temporal (HCV4d) aspects of their transmission history, and frame them in their global context. Further, our results highlight how the patterns of virus flow revealed by statistical phylogenetic and phylogeographic analyses can assist in prioritizing interventions.

## Methods

### Study patients

The study included 245 Saudi Arabian nationals who were chronically infected with HCV as determined by anti-HCV serology and a detectable HCV RNA for more than six months. The patients were recruited between 2007 and 2014 from three major hospitals in Riyadh city including the King Faisal Specialist Hospital & Research Center, the Prince Sultan Military Medical City, and the King Khalid University Hospital. The study was approved by the institutional review board of all participating hospitals (Project # RAC 2090001), and conducted in accordance with the Helsinki Declaration of 1975. Informed consents were obtained from all patients.

### Sequence dataset compilation

New E1 gene region sequence data (H77 nucleotide positions 914–1490) were complemented with a selection of publicly available sequences to assemble the most comprehensive dataset for investigating the evolutionary history of HCV4a and HCV4d. The new sequence data are available from Genbank under accession numbers KY655493-KY655737. Further methodological details are provided in Supplementary Information.

### Timed phylogenetic inferences

Phylogenetic trees were estimated using the Bayesian evolutionary analysis by sampling trees (BEAST) software^14^. Because accurate evolutionary rates could not be estimated based on the differences in sampling date of the HCV4a isolates, we capitalized on previously published data with strong temporal signal to calibrate the molecular clock^15^ (see Supplementary Information for details). We specified a codon position partitioning model (the SRD06 model^16^) for both subtype data sets. A flexible non-parametric model^17^ was fitted to describe demographic changes for HCV4d, but this parameter-rich model could not be informed by the HCV4a data. Because preliminary analysis showed that Egypt is the source of most HCV4a lineages, and Egyptian data dominate the HCV4a dataset (Table 1), we follow Pybus, *et al*.^18^ and resorted to a constant-logistic (con-log) growth model for which we needed to specify an informative prior distribution on the t_50_ parameter. The con-log model assumes a period of exponential growth (starting at the time of the most recent common ancestor (t_MRCA_)) that is preceded by and stabilizes into a phase with constant population size. In this model t_50_ represents the point in time where the population size is half the size of the (constant) population size at present. Our prior choice on t_50_ was informed by the timing of the parenteral anti schistosomiasis (PAT) campaigns in Egypt^10^. Specifically, we centered t_50_ at 1975 and specified the standard deviation on the normal distribution such that the 2.5 and 97.5 percentiles of the distribution correspond to 1961 to 1989.

We obtained estimates of the posterior distribution using Markov chain Monte Carlo (MCMC) sampling and ran several independent MCMC analyses that were combined after removal of the burn-in and visual inspection of convergence and mixing using Tracer v1.6.0 (http://tree.bio.ed.ac.uk).

### Phylogeographic inference

Prior to extensive phylogeographic reconstructions, we tested whether the data contain signal that can inform phylogeographic model parameter estimations, and assessed which model is most appropriate for describing the patterns of spread (see Supplemenraty Information for details). Following these results we adopt a model that allows for different rates to and from a particular location^19^. To identify the subset of migration rates that is most informative to reconstruct the phylogeographic history we combine this model with the Bayesian stochastic search variable selection (BSSVS) procedure and use SpreaD3^20^ to calculate Bayes factor (BF) support for all possible types of location exchanges. Estimates of the posterior expected number of migration events between all pairs of locations (Markov jumps) were computed through stochastic mapping techniques^21^,^22^. All phylogeographic reconstructions were run on empirical tree distributions obtained from the timed phylogenetic inferences^23^.

### Phylogeographic rarefaction curve

While a visual inspection of a phylogeographic tree may provide indications of the migration intensity, it may provide little insight to what extent this is sensitive to the sampling. It also does not scale well to larger sets of trees such as those now routinely obtained using Bayesian phylogenetics. We tackle this with what we refer to as the phylogeographic rarefaction curve, which plots the expected number of introduction events into a specific location (y-axis) as a function of the number of taxa randomly selected from all taxa sampled at that location (x-axis). For this we implemented a script that uses functions from several R packages^24^,^25^,^26^. The y-axis values for the Saudi Arabia HCV4a epidemic were computed by repeating the procedure we detail in Supplementary Information for 100 random subsamples of size *n*, with 1 ≤ *n* ≤ N and N the total number of Saudi Arabian taxa.

## Results

### Sequence datasets

An overview of the dataset composition is provided in Table 1. The complete sequence datasets of HCV4a and 4d contained 568 and 120 taxa respectively. There were substantially more publicly available sequences from non-Saudi Arabia locations for HCV4a (n = 433) than for HCV4d (n = 9). These taxa were from 13 and 6 countries for the HCV4a and HCV4d dataset respectively. Most of the non-Saudi Arabia HCV4a taxa are from Egypt (n = 366), which is not surprising because of a history of explosive spread of this subtype in Egypt^18^.

Initial analyses of both datasets revealed that a small number of taxa clustered as an outgroup separated by long branches. Because this study focused on the more recent phylogeographic events these were removed from the dataset, leaving 555 taxa for HCV4a and 114 taxa for HCV4d. As only a limited number of HCV4a taxa were sampled in countries other than Egypt or Saudi Arabia (10.8%), these were grouped under a single location state (‘other’) in the phylogeographic analyses.

### Subtype 4a

**Figure 1:**
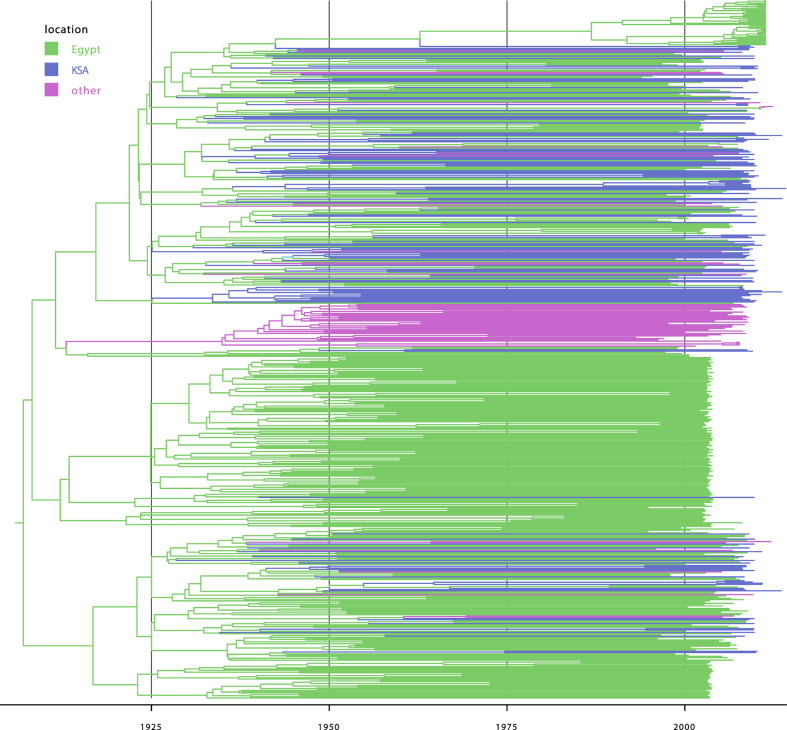
Bayesian maximum clade credibility tree of the HCV4a dataset. Tips and internal branches are colored according to the most probable reconstructed ancestral state (location). The correspondence between the colors and locations is as in the legend.

Our investigation of the epidemiological relationships of HCV4a between Saudi Arabia, Egypt and the ‘other’ locations using phylogeographic methods confirms a dominant role for Egypt in the HCV4a spread. Specifically, the posterior inclusion probabilities of the virus migration rates from Egypt to Saudi Arabia and to the ‘other’ locations were indistinguishable from 1, which is reflected in the very high BF support for these rates of ≥1105. None of the other migration rates was well supported (BF support <3). We also quantified the expected number of migration events between all possible pairs of locations (Markov jumps, Table 2). This corroborates what is evident from Fig. 1, namely that Egypt is the most important source location, and most of the movements out of Egypt (83.73%) are to Saudi Arabia. To some extent, migration out of Saudi Arabia also occurs, but this involves only a small fraction of all virus movements. The fact that only few migrations are directed towards Egypt further highlights Egypt as a net exporter in the history of HCV4a.

**Figure 2:**
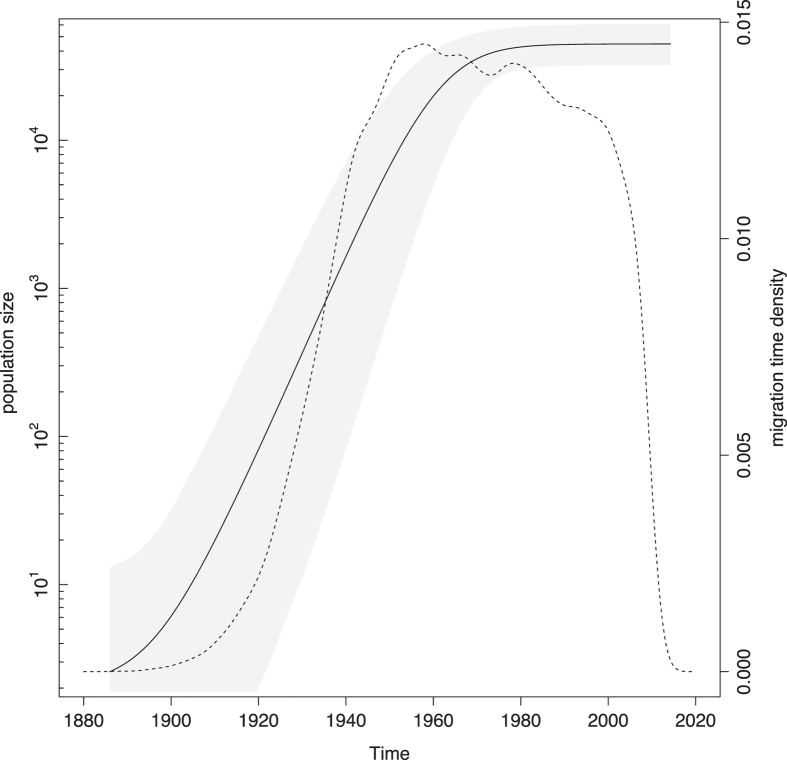
Combined plot of HCV4a population size growth and migration time density. The solid black line represents the estimated product of the effective population size and generation time (*N*_e_*tau) trajectory on a log_10_ scale (left axis), which reflects the number of individuals that contribute offspring to the descendent generation. The grey-shaded area marks the associated 95% HPD interval. The striped line shows the migration density (right axis). After a steep increase in the number of migrations in the period between 1920 and 1950, the migration intensity remained fairly steady.

Although reconstructing the HCV4a demographic history is not the focus of this work, it is reassuring that the estimated increase in population size precedes the migration density through time (Fig. 2).

### Visualizing the transmission network size structure

**Figure 3:**
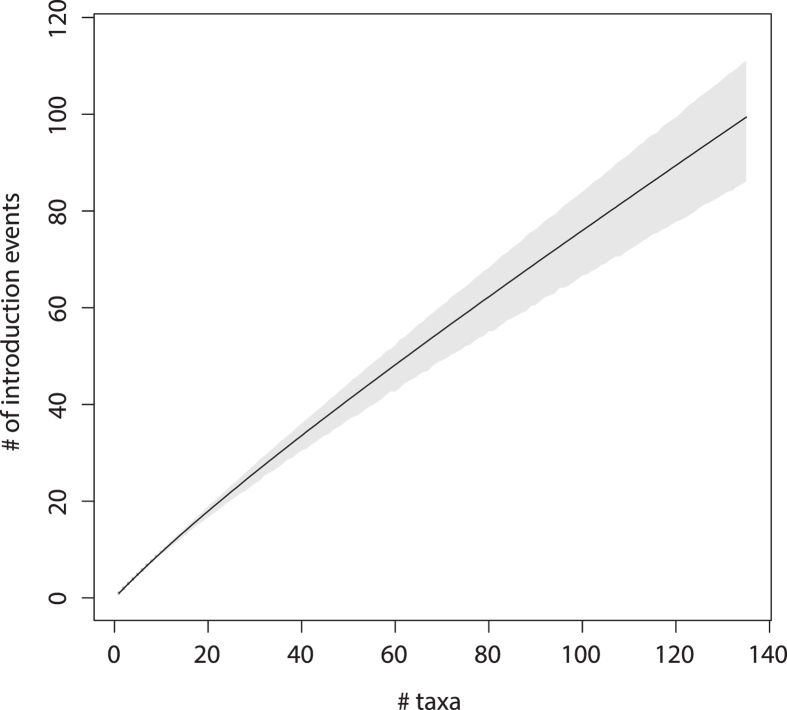
Phylogeographic rarefaction curve. The solid line represents the expected number of introduction events in Saudi Arabia for *n* randomly chosen Saudi Arabian taxa. The grey-shaded area marks the 95% credible interval.

We built a phylogeographic rarefaction curve to determine how the Saudi Arabia HCV4a epidemic is shaped by cross-border migration (Fig. 3). Because each introduction event marks the start of a local transmission network, and the probability of detecting a previously unrecognized introduction event hence depends both on the number of local transmission networks in the total sample and their relative abundance, the gradient of the slope of such curves has an epidemiologically relevant and intuitive interpretation. For example, if the sample consists of a few larger clusters complemented with many smaller ones, the curve is expected to have a mild slope because most of the additional isolates represent the already detected larger transmission networks, and vice versa.

The detection of new subpopulations at a high rate (Fig. 3) reveals that the Saudi Arabia HCV4a epidemic consists of many similar-sized small transmission clusters rather than being dominated by one or a few large local circulation networks. This agrees with the observation that only 32/135 Saudi Arabian taxa group in 14 well-supported clusters (posterior root node support ≥0.95) of size 2, 3 or 5 in the maximum clade credibility (MCC) summary tree (Fig. 1). Of these, only 6 transmission pairs likely represent recent transmission events (t_mrca_ ≤ 10 years and maximum within cluster pairwise genetic distance ≤1%). The fragmented nature of the epidemic is also reflected by the high number of introductions into Saudi Arabia (centered around 99.4, Supplementary Information, Figure S1). Further, the constancy of the slope indicates that the current sampling only reveals a restricted number of introduction events.

### Subtype 4d

**Figure 4:**
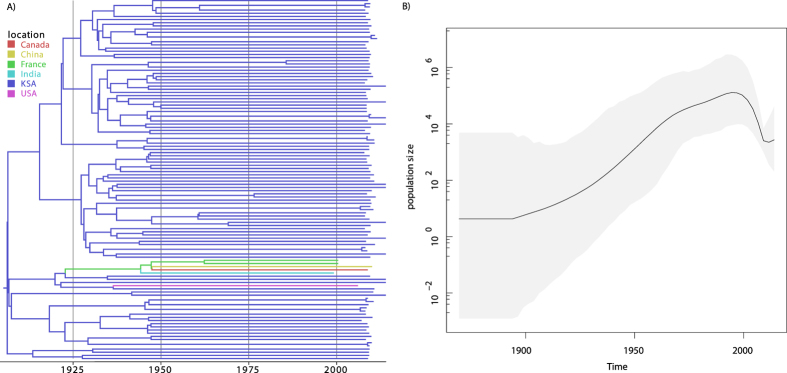
Left panel: Bayesian maximum clade credibility tree of the HCV4d dataset. Tips and internal branches are colored according to the most probable reconstructed ancestral state (location). The correspondence between the colors and locations is as in the legend. Right panel: Plot of the demographic history for HCV4d. The black line represents the change in mean effective number of infections through time on a log_10_ scale. The surrounding grey shaded area indicates the associated 95% HPD interval. The mean estimate is largest in 1994 and the timings of the maximum values for the upper and lower boundaries of the 95% HPD interval are 1992 and 1997.

Unlike for HCV4a, the historical epidemic dynamics of HCV4d are not well characterized. We therefore used our Saudi Arabia-focused sample to estimate changes in viral population size through time (Fig. 4). In contrast to the HCV4a dataset there was a sufficient accumulation of evolutionary divergence over the sampling time period to calibrate the molecular clock. The mean evolutionary rate was estimated at 9.57 × 10^4^ (95% Highest Posterior Density (HPD) interval: 6.38–13.0 × 10^4^), which closely matches the subtype 1a and 1b E1 rate estimate (Supplementary Information, Table S1), and reassures that the latter are appropriate external calibrations to estimate timings of past evolutionary and epidemiological events. The plot of changes in population size through time (Fig. 4, right panel) reveals an approximately exponentially growing population from the beginning of the 20^th^ century to about 1967, followed by a period of less rapid growth up to the beginning of the sampling time period.

## Discussion

We made use of statistical phylogenetics tools to investigate the epidemiological history of the dominant HCV subtypes in Saudi Arabia using newly generated virus genetic data. The availability of sufficient HCV4a reference sequences enabled us to directly investigate the patterns of virus flow relating Saudi Arabia to other locations, Egypt in particular. For HCV4d on the other hand, no representative reference data are currently available which is why we focused on the temporal aspects of the transmission dynamics for this subtype.

Our results show that recent HCV4a transmissions between countries likely have an origin in Egypt (Table 2), which is in line with previous findings concerning a central role of this country in the global HCV4a epidemic^27^,^28^,^29^. Virus spread between Saudi Arabia and Egypt is predominantly directed towards Saudi Arabia, showing that both HCV4a epidemics are connected through a source-sink relation, perhaps linked to the large flow of Egyptian migrant workers^13^. The virus is also transmitted from Egypt to countries from geographically diverse locations that were grouped in the ‘other’ category (Table 1). Saudi Arabia plays a limited secondary role in HCV4a exportation and viral flow out of the country is only partly directed to Egypt (Table 2). This can point to limited remigration of Egyptian migrant workers or it can be related to the large Asian and other African communities in Saudi Arabia and to the large numbers of foreign pilgrims that visit the country^30^,^31^. Unfortunately, the lack of a comprehensive geographic sampling and the focus on a limited genome region prevented us from directly evaluating this hypothesis and from corroborating previously found links. For example, migrant workers have also been invoked to account for the local westward spread of HCV4a out of Egypt^27^, but our sampling does not include Lybian nor Tunesian isolates. Likewise, Egyptian immigrants have been linked to the spread of HCV4a to Europe^28^,^29^.

We also found that the large number of introduction events shapes the structure of the Saudi Arabia HCV4a epidemic (Fig. 3), for which we rely on a transmission cluster analysis using phylogenetic trees. Because the sampling density impacts the extent of clustering, this provides a lower boundary on the introduction count. The large number of introduction events into Saudi Arabia translates to many independent local transmission networks. Most of these are single lineages, and of the remainder only six pairs represent well-supported recent transmission links. Combined, these results suggest limited onwards HCV4a transmission within Saudi Arabia and promote the targeted screening and treatment of (former) immigrant workers as an effective means to lessen the HCV4a burden.

HCV sequence datasets often lack a clear temporal signal, making it common practice to calibrate the molecular clock with other empirical evolutionary rate estimates (e.g. this study and refs 9, 18, 32, 33, 34, 35, 36). These are usually not from the same genotype or subtype (e.g. this study and refs 9,18,42,33, 34, 35), which might raise questions about their suitability. Our finding that the subtype 4d substitution rate - obtained under a tip-date informed model - closely matches that of the frequently used estimates reported by Pybus, *et al*.^35^ and Gray, *et al*.^37^ (Table S1), offers reassurance that these external calibrations are appropriate to estimate timings of past evolutionary and epidemiological events. Nonetheless, it remains important to keep in mind that substitution models may not perfectly correct for multiple substitutions, and their effect will manifest more rapidly on divergence time estimates based on faster-evolving regions compared to more conserved genomic regions. Because the reported divergence timings are based on the rapidly evolving E1 gene region^37^, they represent lower bounds. More accurate results could of course be obtained by longer genomic markers, preferably complete genome sequences. Of note, the highly similar evolutionary rates result in largely overlapping estimates of the subtype 4a (1905, 95% HPD: 1885–1926) and 4d (1907, 95% HPD: 1872–1943) t_MRCA_. These timings are compatible with a recent origin of genotype 4 (1733, 95% HPD 1650–1805, ref. 9), and show it took only around 170 years for genotype 4 viruses to cross the continent.

Previous phylogenetic analyses have demonstrated that HCV genotype 4 emerged in Central Africa, from where it spread to other regions^9^,^38^. The current genotype 4 prevalence and geographic distribution likely still reflects to a large extent the early historical diversification and dissemination events, with Central and Eastern sub-Saharan Africa and the Middle East now experiencing the highest burden^1^. The absence of subtype 4d in surveillance studies in West Africa^38^,^39^,^40^ and Central Africa^9^,^41^,^42^,^43^ suggests that this subtype may have emerged along a more eastward trajectory. Although subtype-level data from this region are scarce, the detection of HCV4d in 22% of seropositive samples in a small-scale Ethiopian survey^44^ supports such a scenario, as do the likely introduction of this subtype in southern Italy by returning colonists, of whom 82% were stationed in the Horn of Africa (Eritrea, Ethiopia and Somalia, the others inhabited Libya)^45^, and its high prevalence in Saudi Arabia^12^,^13^.

The likely eastward spread of subtype 4d to the Middle East may be linked to human mobility, as is usually the case for virus dispersal^6^,^32^,^46^,^47^. This variant is rarely reported (<1%) in the well-surveyed Egyptian epidemic^13^, but the large population of migrant workers from the Horn of Africa^30^,^31^ may have facilitated HCV4d dispersion in a similar way as for HCV4a from Egypt. Therefore, whereas the Saudi Arabian samples dominate the subtype 4d dataset, the reconstructed HCV4d demographic history (Fig. 4) may at least partly reflect the regional epidemiological dynamics. Although it remains difficult to determine the precise extent of this, the expectation that incidence trends will be reflected in effective population size patterns^48^,^49^,^50^,^51^, often allowing demographic trends to be linked to important societal changes^18^,^35^,^36^,^52^,^53^, may help discriminate between competing hypotheses of the dominant source(s) of transmissions. The absence of consistent screening of blood donors in African countries other than Zimbabwe and South Africa before 2002^54^, and the start of the World Health Organisation’s programme to reduce the transmission of blood-borne pathogens through the promotion of safe injection practices (the SIGN initiative) in 1999^55^, which occurred after the start of the decline of the HCV4d effective population size (Fig. 4), indicate that unsafe injection practices and blood transfusions in countries from the Horn of Africa may not explain the observed temporal pattern. On the other hand, the declining subtype 4d epidemic coincides remarkably well with the period of increasing awareness for safe clinical practices and the start of the screening for HCV in blood products in Saudi Arabia^56^,^57^. Together, these indirect lines of evidence suggest that our subtype 4d sample to a large extent reflects the Saudi Arabian epidemic, which in turn points towards a limited importation of HCV4d viruses through migrant workers from the Horn of Africa. Further, as the timing of the decrease in effective population size does not match with a concomitant increase in substance abuse in Saudi Arabia^56^, the pre-1994 expansion was most likely not heavily impacted by injecting drug use (IDU). Nowadays, good screening practices impede the spread of HCV in Saudi Arabia through infected blood products^56^,^57^, and sporadic transmission through various modes, including but not limited to IDU and needle-stick injuries^13^, maintain the spread of HCV. We note, however, that our subtype 4d dataset only offers limited insights and additional sampling efforts, in particular in the Horn of Africa (Sudan, Ethiopia, Eritrea and Somalia), are needed to shed light on the spatial dispersal patterns. Additional sampling is also required to evaluate the role of Saudi Arabia in the global HCV4d spatial circulation, and may prove useful in establishing the presence and nature of links with the 4d epidemics in Turkey^58^ and with the European 4d epidemic among injecting drug users and HIV-1 co-infected MSM^29^,^59^.

As noted by Iles, *et al*.^9^, the presence of other subtypes in a region (e.g. subtypes 4n, 4m, 4l, 4r and 4o in Saudi Arabia^13^ and 4f, 4l, 4m, 4n and 4o in Egypt^60^) indicates that among all variants introduced, HCV4d was the one that by chance was able to benefit most from changes in human behaviour in Saudi Arabia, as was the case for HCV4a in Egypt. The remarkable difference in absolute scale of the HCV epidemics in both regions, with prevalences of respectively ~1.5% in Saudi Arabia versus ~15% in Egypt^1^, is likely explained by the PAT campaigns that amplified the Egyptian subtype 4a^10^,^11^ but not the subtype 4d reservoir. While the subtype 4a growth plateaus from the 1980s onwards, the growth period of subtype 4d extends into the 1990s (Figs 2 and 4). Because informative prior distributions were specified for the substitution rate and the t50 parameter of the coalescent model for the subtype 4a dataset, we refrain from speculating on possible causes that can underlie this difference.

In conclusion, our analyses reveal tight links between the Saudi Arabia HCV4a epidemic and the one in Egypt, indicating that intervention and treatment efforts directed against HCV4a should focus on immigrant populations from Egypt. In contrast, the HCV4d epidemic may be more locally confined, which implies that public health efforts targeting domestic modes of transmission may be more efficient in this case. This study is a powerful example of the value for public health planning of the evolutionary analysis of temporally and geospatially referenced virus genetic data, which are becoming increasingly available owing to upscaled genotyping efforts that accompany the roll out of direct acting antivirals based therapies^61^.

## Additional Information

**How to cite this article:** Al-Qahtani, A. A. *et al*. The epidemic dynamics of hepatitis C virus subtypes 4a and 4d in Saudi Arabia. *Sci. Rep.* **7**, 44947; doi: 10.1038/srep44947 (2017).

**Publisher's note:** Springer Nature remains neutral with regard to jurisdictional claims in published maps and institutional affiliations.

## Supplementary Material

Supplementary Information

## Figures and Tables

**Table 1:** Overview of the sequence dataset composition.

	HCV4a	HCV4d
Canada	14 (3)	2 (1)
China	—	1
Cyprus	6	—
DRC	2 (2)	—
Denmark	2	—
Egypt	366 (3)	3 (3)
France	1	2
India	—	1
Japan	1	—
Saudi Arabia	135*	110* (2)
Pakistan	2	—
Portugal	24 (1)	—
South Africa	2 (2)	—
Sri Lanka	2 (1)	—
USA	10	1
Uganda	1 (1)	—
*total*	568 (13)	120 (6)

The number of sequences per location is given for both subtypes. Numbers between brackets indicate how many sequences of that location did not cluster in the diversity of interest, and were not retained in the final dataset (see main text). The multiple sequence alignments for HCV4a and HCV4d span 576 nt (corresponding to H77 nt positions 914–1490). The HCV4a isolates were collected between 2000 and 2014, and those for HCV4d between 2002 and 2014. *All Saudi Arabia sequences were newly generated for this study.

**Table 2:** Posterior probabilities for all possible types of migration events.

location		posterior probability (%)
from	to	
Egypt	Saudi Arabia	79.12
Egypt	other	15.37
Saudi Arabia	Egypt	1.95
Saudi Arabia	other	2.96
other	Egypt	0.46
other	Saudi Arabia	0.15
