# Supplementary material for: The epidemic dynamics of hepatitis C virus subtypes 4a and 4d in Saudi Arabia
Source: Sci Rep. 2017 Mar 21;7:44947. doi: 10.1038/srep44947 (PMC5359580; doi:10.1038/srep44947)
Supplement: Supplementary Information [file srep44947-s1.doc]

**Supplementary information for “The epidemic dynamics of hepatitis C virus subtypes 4a and 4d in Saudi Arabia”.**

Ahmed A. Al-Qahtani, Guy Baele, Nisreen Khalaf, Marc A. Suchard, Mashael R. Al-Anazi, Ayman A. Abdo, Faisal M. Sanai, Hamad I. Al-Ashgar, Mohammed Q. Khan, Mohammed N. Al-Ahdal, Philippe Lemey and Bram Vrancken

Table of Contents

Extraction of RNA and cDNA synthesis [2](#__RefHeading___Toc327628674)

HCV E1 polymerase chain reaction (PCR) [2](#__RefHeading___Toc327628675)

DNA sequencing [3](#__RefHeading___Toc327628676)

Genotyping of HCV [3](#__RefHeading___Toc327628677)

Sequence dataset compilation [3](#__RefHeading___Toc327628678)

The prior distribution on the mean evolutionary rate parameter [4](#__RefHeading___Toc327628679)

Phylogeny-trait correlations [5](#__RefHeading___Toc327628680)

How the phylogeographic rarefaction curve is built [6](#__RefHeading___Toc327628681)

Table S1: Overview of the HCV subtype 1a and 1b substitution rate estimates [7](#__RefHeading___Toc327628682)

Table S2: Overview of the model comparison results [8](#__RefHeading___Toc327628697)

Figure S1: Posterior probability of the number of introductions into the KSA for subtype 4a [9](#__RefHeading___Toc327628698)

References [10](#__RefHeading___Toc327628699)

## Extraction of RNA and cDNA synthesis

Viral RNA was extracted from 200 µl of each serum sample using the QIAmp® MinElute™ Virus Spin Kit (QIAGEN, CA, USA) according to the manufacturer’s instructions and recommendations. 10 µL of extracted RNA was mixed with 1 µL of random hexamers (50 ng/ µL) in 1.5 ml eppendorf tube and heated at 65^0^C for 15 min and cooled down at room temperature for 5 min. This was followed by the addition of 5 µL of 5X cDNA synthesis buffer, 2.0 µL of 10 mM dNTP mix, 1 µL of 0.1M DTT, 1 µL RNase inhibitor (40U/µL), 5 Units of cloned AMV reverse transcriptase and DEPC water to a total volume of 25 µL. The mixture was incubated for 1 hr at 50 ^0^C and the reaction was terminated by incubating at 85 ^0^C for 5 min and stored at -70 ^0^C until used.

## HCV E1 polymerase chain reaction (PCR)

PCR was performed in 25-µL reaction volumes. The mixture contained 2X GoTaq Green Master Mix (Promega, Wisconsin, USA), 3 µL template cDNA, 25 pmol of the HCVE1FOR forward primer (5'-TGGGTAAGGTCATCGATACC-3') and HCVE1REV reverse primer (5’-TGCCARCTBCCRTTGSTRTT-3’). PCR conditions were as follows: 95°C for 2 min (initial denaturation), followed by 35 cycles of denaturation at 94°C for 1 min, annealing at 55°C for 1 min, extension at 72°C for 1 min, and a final extension of 5 min at 72°C. The PCR products were then visualized in 1.5% agarose gel stained with 1 μg/ml Ethidium Bromide.

## DNA sequencing

PCR products were gel purified and sequenced using automated DNA sequencing system (ABI 3100) and BigDye® Terminator v3.1 cycle sequencing Kit (Applied Biosystems, Foster City, CA, USA) according to the manufacturer's instructions. The integrity of the sequence chromatograms of full-length of E1 gene was checked in SeqMan Pro module of DNASTAR (DNASTAR, Inc., Madison, WI, USA). Sequences were assembled and edited using the LaserGene suite of DNASTAR software [^1^](#_ENREF_1).

##

## Genotyping of HCV

Nucleic acids were extracted from sera using the QIAmp MinElute Virus Spin Kit (QIAGEN, Santa Clarita, California, USA) following the manufacturer’s instructions. Genotyping was carried out using INNO-LiPA HCV II (Innogenetics NV, Gent, Belgium) according to the manufacturer's instructions.

## Sequence dataset compilation

We followed the strategy of [Graf, et al^. 2^](#_ENREF_2) to compile the most comprehensive dataset for investigating the evolutionary history of HCV subtypes 4a and 4d. This resulted in two datasets, one per subtype, which were analysed separately. Specifically, we retrieved the 500 best hits from Genbank (downloaded in April 2015) in a local blastn search for all newly obtained sequences. Duplicate hits were filtered and from the unique hits we only kept those sequences with a) known sampling location, b) at least 75% sequence overlap, c) a genotype classification confirmed by COMET [^5^](#_ENREF_5) and d) a known sampling date or a reference in the Genbank record to an article publication or Genbank modification date. Sequences were aligned with Muscle [^6^](#_ENREF_6) and edited with Aliview [^7^](#_ENREF_7).

## The prior distribution on the mean evolutionary rate parameter

Because HCV sequence datasets often lack a clear temporal signal it is common practice to use other empirical evolutionary rate estimates for specifying a suitable prior distribution on the evolutionary rate parameter (e.g. [^8-13^](#_ENREF_8)). Here, we estimate gene-specific evolutionary rates using the data from [Gray, et al^. 14^](#_ENREF_14). We follow [Gray, et al^. 14^](#_ENREF_14) in using the non-parametric Bayesian Skyline coalescent model and fitting an HKY nucleotide substitution model [^17^](#_ENREF_17). The evolutionary rate was estimated under an uncorrelated clock model with the rates drawn from a lognormal distribution [^18^](#_ENREF_18). We partitioned the genome by gene and accommodated evolutionary rate variation among genes (using a relative rate approach, [^19^](#_ENREF_19)). We specified hierarchical prior distributions over the gene-specific evolutionary parameters to share information across the gene partitions [^20^](#_ENREF_20). We jointly summarized the posterior distributions for the HCV 1a and 1b E1 evolutionary rate and used this to specify a normal prior distribution on the mean evolutionary rate for our 4a data set. Specifically, the mean of this prior distribution was set to the mean of the combined posterior density and the standard deviation was set so that the 2.5 and 97.5 percentiles of the normal prior corresponded to those of the combined posterior density. The BEAST XML files used for estimating the gene-specific evolutionary rates are available from the authors upon request, and the gene-specific evolutionary rate estimates are listed in Table S1.

## Phylogeny-trait correlations

Before estimating the history of spread we first confirmed that neighboring taxa are more likely to share the same location state than would be expected by chance alone (i.e. we measured the support for phylogenetic structure of the (discrete) location trait). We also tested whether the data better support a (more realistic) model in which rates can vary according to the direction of movement (an nonreversible model, [^21^](#_ENREF_21)) over a model in which viruses move at the same rate to and from a location (a reversible model, [^22^](#_ENREF_22)) for describing the process of geographical spread. For this we follow the approach described by [Graf, et al^. 2^](#_ENREF_2). Briefly, the former entails testing whether the null hypothesis of no phylogenetic correlation structure among the traits can be rejected by contrasting marginal likelihoods for a trait diffusion model on an inferred maximum clade credibility (MCC) tree versus a starlike tree. For this comparison as well as for testing the reversible versus the nonreversible diffusion model, we used the stepping stone marginal likelihood estimator implemented in BEAST [^23-25^](#_ENREF_23). The (log) marginal likelihood estimates can be used to calculate (log) Bayes factors (BFs) in support of one model over the other.

In agreement with the apparent almost unidirectional flow from Egypt to other locations (see Figure 1 in main text), the model fit comparisons (Table S2) reveal a strong support for the phylogeographic model that allows for different rates depending on the direction of movement (log BF = 96.17). Under this model, we found strong support for clustering by location (log BF = 177.2).

## How the phylogeographic rarefaction curve is built

We quantify the introductions by traversing trees direction tip-to-root starting from a Saudi Arabian tip, and track the location state at each next internal node. Nodes are added to the path of interest as long as the virus is inferred to have resided in Saudi Arabia (e.g. until an introduction event is detected). Next, we determine the location trajectories to all descendant Saudi Arabia tips from the deepest node in the path of interest to ascertain whether or not they belong to the transmission network that ignited with the detected introduction event. This procedure is then repeated for the next Saudi Arabian tip that is not part of an already encountered transmission cluster until all tips in the current random subsample are screened. To account for the uncertainty in the phylogenetic reconstruction we scan a random subset of 250 trees from the posterior distribution for each subsample. Note that this approach can be generalized to any discrete trait of interest (e.g. risk group, age category, …).

## Table S1: Overview of the HCV subtype 1a and 1b substitution rate estimates. The HCV 1a and 1b full genome sequence data sets from [^14^](#_ENREF_14) were used to estimate gene-specific evolutionary rates. The substitution rate is expressed as the number of nucleotide substitutions (x10^-3^) per site per year. The 95%HPD interval is given between brackets.

| gene | subtype | |
| --- | --- | --- |
|  | 1a | 1b |
|  |  |  |
| Core | 0.79 (0.53-1.02) | 0.48 (0.28-0.72) |
| E1 | 1.23 (0.90-1.58) | 0.97 (0.52-1.36) |
| E2 | 2.17 (1.57-2.79) | 2.41 (1.27-3.25) |
| P7 | 2.18 (1.41-3.08) | 1.15 (0.60-1.62) |
| NS2 | 1.41 (0.96-1.81) | 1.15 (0.64-1.62) |
| NS3 | 1.00 (0.73-1.28) | 0.94 (0.53-1.32) |
| NS4A | 0.99 (0.68-1.37) | 1.42 (0.75-2.22) |
| NS4B | 1.06 (0.75-1.37) | 0.98 (0.52-1.38) |
| NS5A | 1.32 (0.97-1.73) | 1.08 (0.63-1.52) |
| NS5B | 1.05 (0.76-1.35) | 0.92 (0.55-1.28) |

## Table S2: Overview of the model comparison results. SS: stepping stone marginal likelihood estimator [^26^](#_ENREF_26). Bayes factors are calculated as the ratio of the marginal likelihoods of both models. Because the likelihoods are expressed as log-likelihoods, this equates to taking the difference of the log-likelihood of competing models.

| model | marginal likelihood estimator SS |
| --- | --- |
| a. symmetric – MCC tree | -327978.59 |
| b. asymmetric – MCC tree | -327882.42 |
| c. symmetric - startree | -328078.48 |
| d. asymmetric - startree | -328059.62 |
| Bayes factor (b – a) | 96.17 |
| Bayes factor (b – d) | 177.2 |

## Figure S1: Posterior probability of the number of introductions into the KSA for subtype 4a, based on the same subset of 250 trees used for Figure 4.

## References

1 Burland, T. G. DNASTAR's Lasergene sequence analysis software. *Methods in molecular biology* **132**, 71-91 (2000).

2 Graf, T. *et al.* Contribution of Epidemiological Predictors in Unraveling the Phylogeographic History of HIV-1 Subtype C in Brazil. *J Virol* **89**, 12341-12348, doi:10.1128/JVI.01681-15 (2015).

3 Camacho, C. *et al.* BLAST+: architecture and applications. *BMC Bioinformatics* **10**, 421, doi:10.1186/1471-2105-10-421 (2009).

4 Altschul, S. F., Gish, W., Miller, W., Myers, E. W. & Lipman, D. J. Basic local alignment search tool. *Journal of molecular biology* **215**, 403-410, doi:10.1016/S0022-2836(05)80360-2 (1990).

5 Struck, D., Lawyer, G., Ternes, A. M., Schmit, J. C. & Bercoff, D. P. COMET: adaptive context-based modeling for ultrafast HIV-1 subtype identification. *Nucleic Acids Res* **42**, e144, doi:10.1093/nar/gku739 (2014).

6 Edgar, R. C. MUSCLE: multiple sequence alignment with high accuracy and high throughput. *Nucleic Acids Res* **32**, 1792-1797, doi:10.1093/nar/gkh340 (2004).

7 Larsson, A. AliView: a fast and lightweight alignment viewer and editor for large datasets. *Bioinformatics* **30**, 3276-3278, doi:10.1093/bioinformatics/btu531 (2014).

8 Iles, J. C. *et al.* Phylogeography and epidemic history of hepatitis C virus genotype 4 in Africa. *Virology* **464-465**, 233-243, doi:10.1016/j.virol.2014.07.006 (2014).

9 Markov, P. V. *et al.* Colonial history and contemporary transmission shape the genetic diversity of hepatitis C virus genotype 2 in Amsterdam. *J Virol* **86**, 7677-7687, doi:10.1128/JVI.06910-11 (2012).

10 Markov, P. V. *et al.* Phylogeography and molecular epidemiology of hepatitis C virus genotype 2 in Africa. *The Journal of general virology* **90**, 2086-2096, doi:10.1099/vir.0.011569-0 (2009).

11 Pybus, O. G. *et al.* Genetic history of hepatitis C virus in East Asia. *J Virol* **83**, 1071-1082, doi:10.1128/JVI.01501-08 (2009).

12 Pybus, O. G., Drummond, A. J., Nakano, T., Robertson, B. H. & Rambaut, A. The epidemiology and iatrogenic transmission of hepatitis C virus in Egypt: a Bayesian coalescent approach. *Mol Biol Evol* **20**, 381-387 (2003).

13 Pybus, O. G. *et al.* The epidemic behavior of the hepatitis C virus. *Science* **292**, 2323-2325, doi:10.1126/science.1058321 (2001).

14 Gray, R. R. *et al.* The mode and tempo of hepatitis C virus evolution within and among hosts. *BMC Evol Biol* **11**, 131, doi:10.1186/1471-2148-11-131 (2011).

15 Drummond, A. J., Nicholls, G. K., Rodrigo, A. G. & Solomon, W. Estimating mutation parameters, population history and genealogy simultaneously from temporally spaced sequence data. *Genetics* **161**, 1307-1320 (2002).

16 Drummond, A. J., Rambaut, A., Shapiro, B. & Pybus, O. G. Bayesian coalescent inference of past population dynamics from molecular sequences. *Mol Biol Evol* **22**, 1185-1192, doi:10.1093/molbev/msi103 (2005).

17 Hasegawa, M., Kishino, H. & Yano, T. Dating of the human-ape splitting by a molecular clock of mitochondrial DNA. *J Mol Evol* **22**, 160-174 (1985).

18 Drummond, A. J., Ho, S. Y. W., Phillips, M. J. & Rambaut, A. Relaxed phylogenetics and dating with confidence. *PLoS Biol* **4**, e88 (2006).

19 Harrison, A. *et al.* Genomic analysis of hepatitis B virus reveals antigen state and genotype as sources of evolutionary rate variation. *Viruses* **3**, 83-101, doi:10.3390/v3020083 (2011).

20 Suchard, M. A., Kitchen, C. M. R., Sinsheimer, J. S. & Weiss, R. E. Hierarchical phylogenetic models for analyzing multipartite sequence data. *Syst Biol* **52**, 649-664 (2003).

21 Edwards, C. J. *et al.* Ancient hybridization and an Irish origin for the modern polar bear matriline. *Current biology : CB* **21**, 1251-1258, doi:10.1016/j.cub.2011.05.058 (2011).

22 Lemey, P., Rambaut, A., Drummond, A. J. & Suchard, M. A. Bayesian phylogeography finds its roots. *PLoS Comput Biol* **5**, e1000520, doi:10.1371/journal.pcbi.1000520 (2009).

23 Baele, G. & Lemey, P. Bayesian evolutionary model testing in the phylogenomics era: matching model complexity with computational efficiency. *Bioinformatics* **29**, 1970-1979 (2013).

24 Baele, G., Li, W. L. S., Drummond, A. J., Suchard, M. A. & Lemey, P. Accurate model selection of relaxed molecular clocks in bayesian phylogenetics. *Mol Biol Evol* **30**, 239-243 (2013).

25 Baele, G. *et al.* Improving the accuracy of demographic and molecular clock model comparison while accommodating phylogenetic uncertainty. *Mol Biol Evol* **29**, 2157-2167 (2012).

26 Xie, W., Lewis, P. O., Fan, Y., Kuo, L. & Chen, M.-H. Improving marginal likelihood estimation for Bayesian phylogenetic model selection. *Syst Biol* **60**, 150-160 (2011).
